# Identification of volatiles from six marine *Celeribacter* strains

**DOI:** 10.3762/bjoc.17.38

**Published:** 2021-02-11

**Authors:** Anuj Kumar Chhalodia, Jan Rinkel, Dorota Konvalinkova, Jörn Petersen, Jeroen S Dickschat

**Affiliations:** 1 Kekulé Institute of Organic Chemistry and Biochemistry, University of Bonn, Gerhard-Domagk-Straße 1, 53121 Bonn, Germany; 2 Leibniz-Institut DSMZ - Deutsche Sammlung von Mikroorganismen und Zellkulturen GmbH, Inhoffenstraße 7b, 38124 Braunschweig, Germany

**Keywords:** GC–MS, isotopes, *Roseobacter*, sulfur metabolism, volatiles

## Abstract

The volatiles emitted from six marine *Rhodobacteraceae* species of the genus *Celeribacter* were investigated by GC–MS. Besides several known compounds including dimethyl trisulfide and *S*-methyl methanethiosulfonate, the sulfur-containing compounds ethyl (*E*)-3-(methylsulfanyl)acrylate and 2-(methyldisulfanyl)benzothiazole were identified and their structures were verified by synthesis. Feeding experiments with [*methyl*-^2^H_3_]methionine, [*methyl*-^13^C]methionine and [^34^S]-3-(dimethylsulfonio)propanoate (DMSP) resulted in the high incorporation into dimethyl trisulfide and *S*-methyl methanethiosulfonate, and revealed the origin of the methylsulfanyl group of 2-(methyldisulfanyl)benzothiazole from methionine or DMSP, while the biosynthetic origin of the benzothiazol-2-ylsulfanyl portion could not be traced. The heterocyclic moiety of this compound is likely of anthropogenic origin, because 2-mercaptobenzothiazole is used in the sulfur vulcanization of rubber. Also in none of the feeding experiments incorporation into ethyl (*E*)-3-(methylsulfanyl)acrylate could be observed, questioning its bacterial origin. Our results demonstrate that the *Celeribacter* strains are capable of methionine and DMSP degradation to widespread sulfur volatiles, but the analysis of trace compounds in natural samples must be taken with care.

## Introduction

**Scheme 1:**
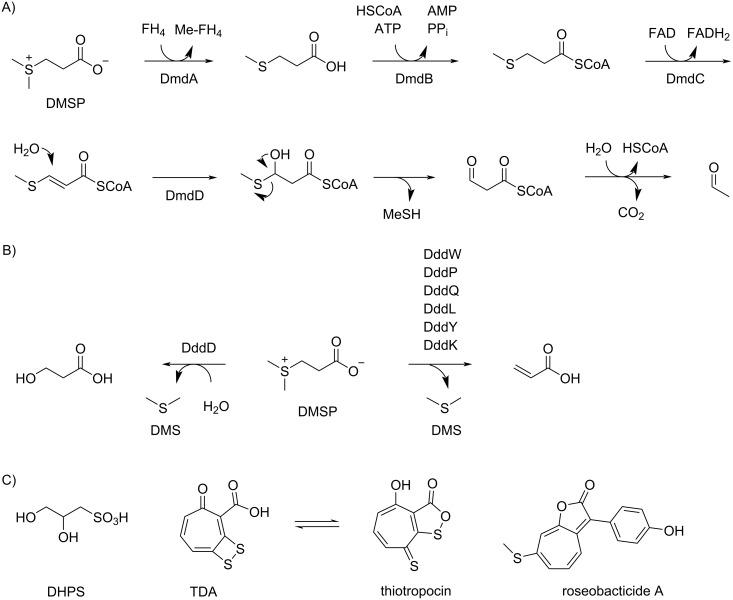
Sulfur metabolism in bacteria from the roseobacter group. A) DMSP demethylation by DmdABCD, B) DMSP hydrolysis by DddP and lysis by DddW, DddP, DddQ, DddL, DddY or DddK, and C) structures of DHPS and sulfur-containing secondary metabolites.

Bacteria from the roseobacter group belong to the most abundant microbial species in marine ecosystems [1–2]. They are present from polar to tropical regions, in marine sediments, in estuarine and open ocean environments in different pelagic zones ranging from surface waters to depths of >2,000 m [3–4]. Some species are associated with other marine organisms, e.g., *Thalassococcus halodurans* DSM 26915^T^ has been isolated from the marine sponge *Halichondria panicea* [5], and *Phaeobacter gallaeciensis* DSM 26640^T^ is an isolate from the scallop *Pecten maximus* [6]. Important interactions are also observed between bacteria from the roseobacter group and various types of marine algae, e.g., the first described organisms *Roseobacter litoralis* DSM 6996^T^ and *R. denitrificans* DSM 7001^T^ were obtained from seaweed [7], while *Dinoroseobacter shibae* DSM 16493^T^ and *Marinovum algicola* DSM 10251^T^ are both isolates from the dinoflagellate *Prorocentrum lima* [8–9]. Especially in algal blooms bacteria of the roseobacter group are highly abundant [10], and here they belong to the main players involved in the enzymatic degradation of the algal sulfur metabolite 3-(dimethylsulfonio)propanoate (DMSP, Scheme 1) [11]. Its catabolism leads either through the demethylation pathway by action of the enzymes DmdABCD to methanethiol (MeSH, Scheme 1A) [12] or through lysis by DddD [13] or hydrolytic cleavage by one of the known DMSP lyases (DddW [14], DddP [15], DddQ [16], DddL [17], DddY [18] or DddK [19]) to dimethyl sulfide (DMS, Scheme 1B).

It has already been pointed out in the 1970s and 1980s that atmospheric DMS is important for the global sulfur cycle [20] and influences the climate on Earth, known as CLAW hypothesis according to the authors’ initials (Carlson, Lovelock, Andreae, Warren) [21], which underpins the relevance of this algal–bacterial interaction. Isotopic labeling experiments demonstrated that also in laboratory cultures roseobacter group bacteria efficiently degrade DMSP into sulfur volatiles [22–23], but also from other sulfur sources including 2,3-dihydroxypropane-1-sulfonic acid (DHPS, Scheme 1C) labeling was efficiently incorporated into sulfur volatiles [24–25]. Notably, DHPS is produced in large quantities by the marine diatom *Thalassiosira pseudonana* [26], and diatoms from this genus live in symbiotic relationship with bacteria of the roseobacter group [27]. Another interesting aspect of sulfur metabolism in marine bacteria from the roseobacter group is the production of the sulfur-containing antibiotic tropodithietic acid (TDA) in *Phaeobacter piscinae* DSM 103509^T^ [28], a compound that is in equilibrium with its tautomer thiotropocin [29] that was first described from *Pseudomonas* sp. CB-104 [30]. Its biosynthesis depends on the clustered *tda* genes [31] and has been studied by feeding experiments with labeled precursors to the wildtype and gene knockout strains of *P. inhibens* DSM 17395^T^, demonstrating the formation of TDA from phenylalanine through phenylacetyl-CoA and the phenylacetyl-CoA catabolon [32–33]. These experiments also led to a suggestion for the mechanism for sulfur incorporation, but further research is required for a deep understanding of TDA biosynthesis. Besides its function as an antibiotic, TDA acts as a signaling molecule, similar to *N*-acylhomoserine lactones, at concentrations 100 times lower than required for a significant antibiotic activity [34]. The biosynthesis of tropone [35] and of the algicidal sulfur-containing roseobacticides [36] are most likely connected to the TDA pathway. Interestingly, in the interaction with marine algae *P. inhibens* can change its lifestyle from a symbiotic relationship during which the antibiotic TDA and growth stimulants are produced to a pathogenic interaction promoted by lignin degradation products in fading algal blooms that induce roseobacticide biosynthesis [36]. All these examples demonstrate the importance of sulfur metabolism for marine bacteria from the roseobacter group. Here we report on the volatiles emitted by six *Celeribacter* species with a special focus on sulfur volatiles. The results from feeding studies with labeled precursors demonstrate that the *Celeribacter* strains can form sulfur volatiles from methionine and DMSP, but also showed that some of the detected sulfur compounds are not or only partly of bacterial origin.

## Results and Discussion

### Headspace analysis

**Figure 1:**
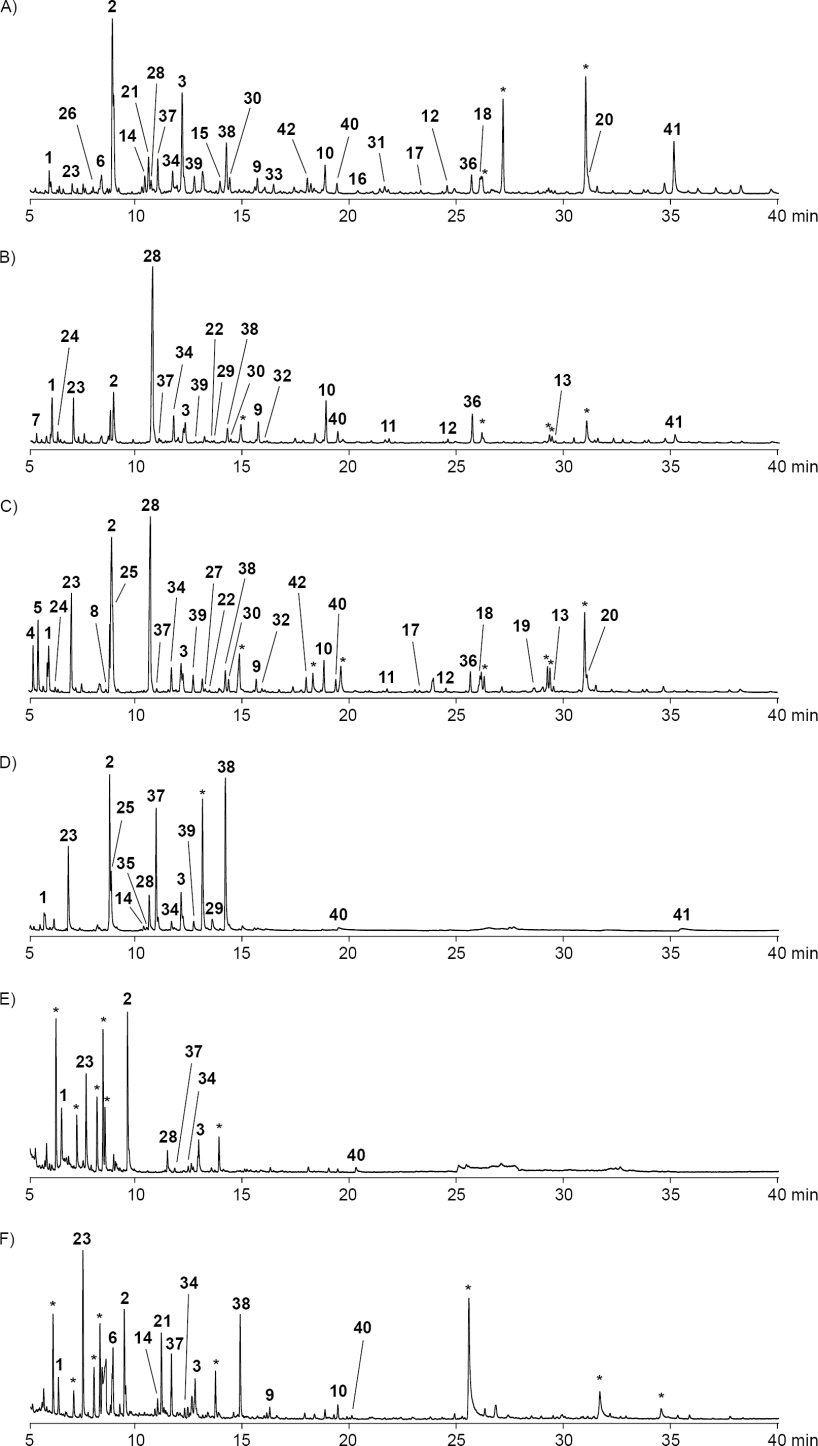
Total ion chromatograms of headspace extracts from A) *C. marinus* DSM 100036^T^, B) *C. neptunius* DSM 26471^T^, C) *C. manganoxidans* DSM 27541^T^, D) *C. baekdonensis* DSM 27375^T^, E) *C. halophilus* DSM 26270^T^, and F) *C. indicus* DSM 27257^T^. Peaks arising from known contaminants are indicated by asterisks.

**Figure 2:**
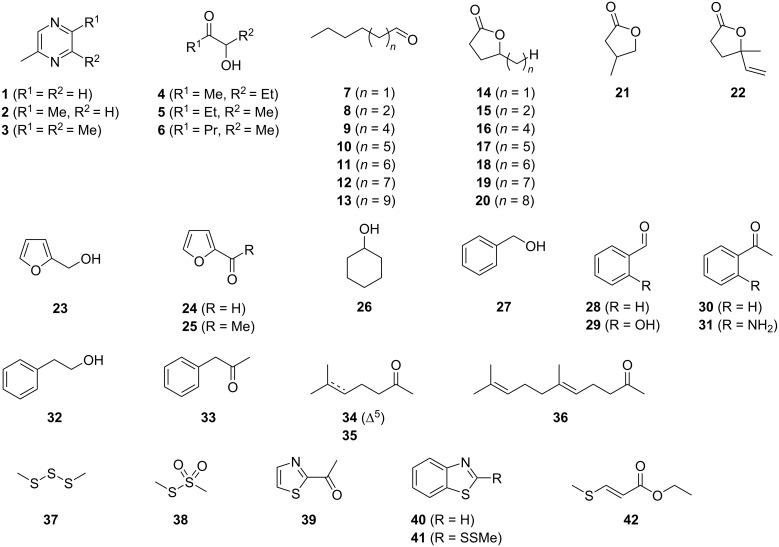
Structures of the identified volatile compounds in the headspace extracts from six *Celeribacter* type strains.

The volatiles released by six marine *Celeribacter* type strains, including *C. marinus* DSM 100036^T^, *C. neptunius* DSM 26471^T^, *C. manganoxidans* DSM 27541^T^, *C. baekdonensis* DSM 27375^T^, *C. halophilus* DSM 26270^T^ and *C. indicus* DSM 27257^T^, were collected through a closed-loop stripping apparatus (CLSA) on charcoal [37]. After extraction with dichloromethane the obtained extracts were analyzed by GC–MS (Figure 1). The compounds were identified by the comparison of the recorded EI mass spectra to library spectra and of retention indices [38] to tabulated literature data (Table 1), or by a direct comparison to authentic standards. The structures of the identified compounds are shown in Figure 2.

**Table 1:** Volatiles from *Celeribacter* spp.

Compound^a^	*I*^b^	*I*(lit.)^b^	Id.^c^	Occurrence^d^					

3-hydroxypentan-2-one (**4**)	812	815 [39]	ri, ms			C			
hexanal (**7**)	813	806 [39]	ri, ms		B				
2-hydroxypentan-3-one (**5**)	818	818 [40]	ri, ms			C			
methylpyrazine (**1**)	831	826 [41]	ri, ms	A	B	C	D	E	F
furfural (**24**)	841	841 [42]	ri, ms		B	C			
furan-2-ylmethanol (**23**)	861	863 [43]	ri, ms	A	B	C	D	E	F
cyclohexanol (**26**)	888	886 [44]	ri, ms	A					
2-hydroxyhexan-3-one (**6**)	899	900 [40]	ri, ms	A					F
heptanal (**8**)	906	901 [45]	ri, ms			C			
2,5-dimethylpyrazine (**2**)	912	908 [45]	ri, ms	A	B	C	D	E	F
2-acetylfuran (**25**)	913	909 [45]	ri, ms			C	D		
pentan-4-olide (**14**)	953	956 [46]	ri, ms	A			D		F
3-methylbutan-4-olide (**21**)	957	958 [47]	ri, ms	A					F
6-methylheptan-2-one (**35**)	959	962 [48]	ri, ms				D		
benzaldehyde (**28**)	961	952 [45]	ri, ms	A	B	C	D	E	
dimethyl trisulfide (**37**)	970	968 [49]	ri, ms	A	B	C	D	E	F
6-methylhept-5-en-2-one (**34**)	988	981 [45]	ri, ms	A	B	C	D	E	F
trimethylpyrazine (**3**)	1000	1000 [45]	ri, ms	A	B	C	D	E	F
2-acetylthiazole (**39**)	1017	1014 [45]	ri, ms	A	B	C	D		
benzyl alcohol (**27**)	1033	1026 [45]	ri, ms			C			
4-methylhex-5-en-4-olide (**22**)	1039	1034 [45]	ri, ms		B	C			
salicylaldehyde (**29**)	1042	1039 [45]	ri, ms		B		D		
hexan-4-olide (**15**)	1052	1056 [50]	ri, ms	A					
*S*-methyl methanethiosulfonate (**38**)	1061	1068 [51]	ri, ms	A	B	C	D		F
acetophenone (**30**)	1065	1059 [45]	ri, ms	A	B	C			
nonanal (**9**)	1103	1100 [45]	ri, ms	A	B	C			F
2-phenylethanol (**32**)	1111	1106 [45]	ri, ms		B	C			
phenylacetone (**33**)	1127	1124 [52]	ri, ms	A					
ethyl (*E*)-3-(methylsulfanyl)acrylate (**42**)	1177	*1144* [53]	ms	A		C			
decanal (**10**)	1203	1201 [45]	ri, ms	A	B	C			F
benzothiazole (**40**)	1221	1222 [54]	ri, ms	A	B	C	D	E	F
octan-4-olide (**16**)	1252	1250 [45]	ri, ms	A					
*o*-aminoacetophenone (**31**)	1292	1296 [55]	ri, ms	A					
undecanal (**11**)	1298	1305 [45]	ri, ms		B	C			
nonan-4-olide (**17**)	1354	1358 [45]	ri, ms	A		C			
dodecanal (**12**)	1400	1408 [45]	ri, ms	A	B	C			
geranylacetone (**36**)	1445	1453 [45]	ri, ms	A	B	C			
decan-4-olide (**18**)	1461	1465 [45]	ri, ms	A		C			
undecan-4-olide (**19**)	1568	1569 [45]	ri, ms			C			
tetradecanal (**13**)	1605	1611 [45]	ri, ms		B	C			
dodecan-4-olide (**20**)	1673	1676 [45]	ri, ms	A		C			
2-(methyldisulfanyl)benzothiazole (**41**)	1860		std	A	B		D		

^a^Identified by GC–MS, known typical contaminants such as plasticizers are not included and all listed compounds were not detected in blank runs with medium plates (except traces of benzaldehyde); ^b^retention index on a HP5-MS GC column and comparison to literature data from the same or a similar type of GC column; ^c^identification based on ri: matching retention index (difference between measured retention index and literature data ≤10 points), ms: mass spectrum matching to a database spectrum, std: direct comparison to an authentic standard; ^d^occurrence in A: *C. marinus* DSM 100036^T^, B: *C. neptunius* DSM 26471^T^, C: *C. manganoxidans* DSM 27541^T^, D: *C. baekdonensis* DSM 27375^T^, E: *C. halophilus* DSM 26270^T^, and F: *C. indicus* DSM 27257^T^.

**Figure 3:**
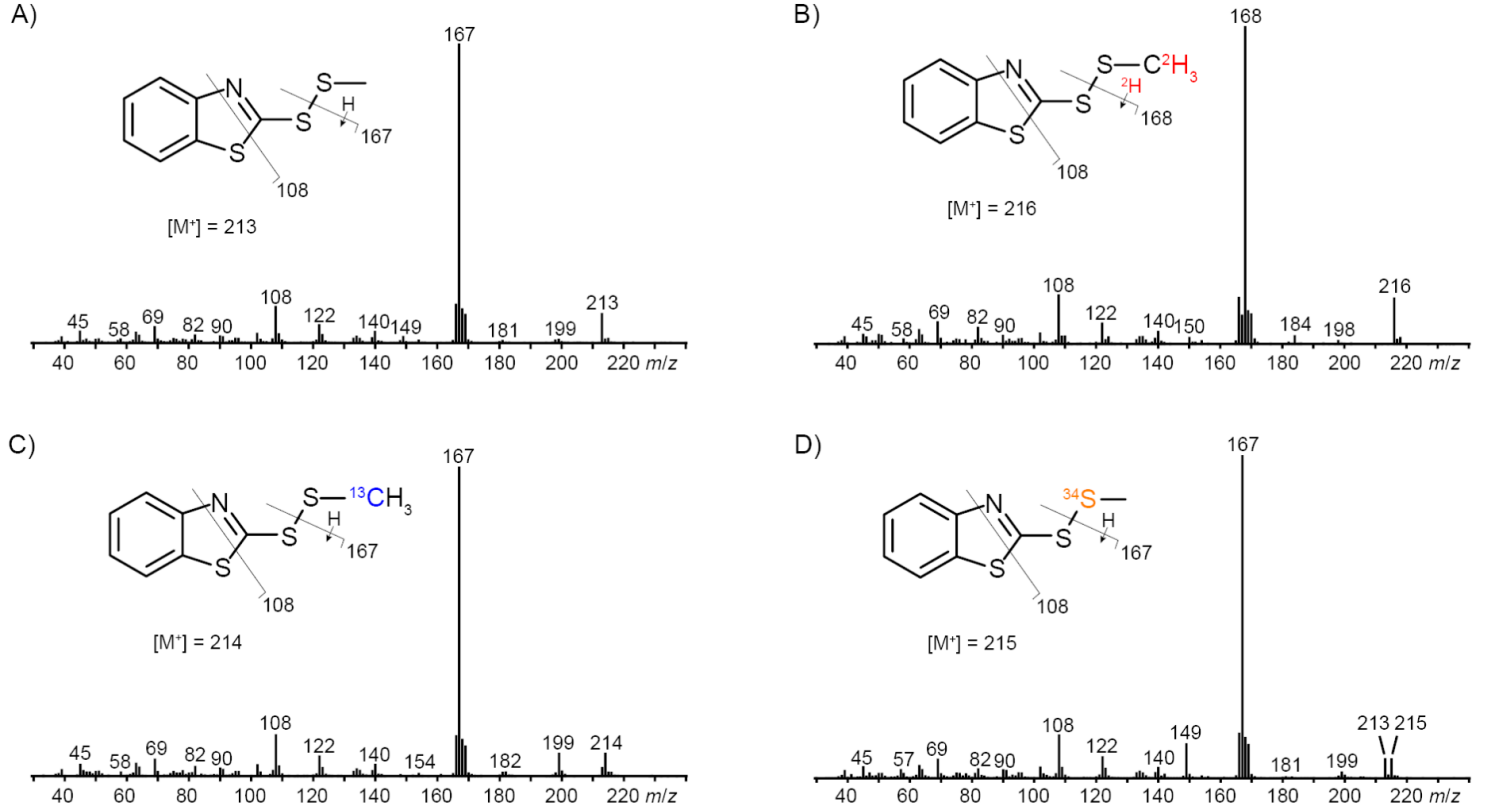
EI mass spectra of A) unlabeled 2-(methyldisulfanyl)benzothiazole (**41**) and of labeled **41** after feeding of B) (*methyl*-^2^H_3_)methionine, C) (*methyl*-^13^C)methionine and D) (^34^S)DMSP.

While the headspace extracts from *C. marinus*, *C. neptunius* and *C. manganoxidans* were particularly rich, the extracts from *C. baekdonensis*, *C. halophilus* and *C. indicus* contained fewer compounds. Most of the observed volatiles are well known [56–57] and were thus readily identified from their mass spectra and retention indices. Pyrazines including methylpyrazine (**1**), 2,5-dimethylpyrazine (**2**) and trimethylpyrazine (**3**) were present in the extracts from all six strains. Notably, also several α-hydroxyketones that have been described as biosynthetic precursors to pyrazines [40], represented by 3-hydroxypentan-2-one (**4**), 2-hydroxypentan-3-one (**5**) and 2-hydroxyhexan-3-one (**6**), were observed in some of the investigated strains. A series of aldehydes ranging from hexanal (**7**) to tetradecanal (**13**) was found in strain specific patterns, with all identified compounds present in the bouquet from *C. manganoxidans*. A similar series of γ-lactones spanning from pentan-4-olide (**14**) to dodecan-4-olide (**20**), in addition to 3-methylbutan-4-olide (**21**) and 4-methylhex-5-en-4-olide (**22**), was detected in strain-specific patterns, with almost all of these compounds present in *C. marinus*; only *C. halophilus* did not emit lactones. Furans included furan-2-ylmethanol (**23**), furfural (**24**), and 2-acetylfuran (**25**). Cyclohexanol (**26**) was observed only once in *C. marinus*, and aromatic compounds included benzyl alcohol (**27**), benzaldehyde (**28**) and salicylaldehyde (**29**), acetophenone (**30**) and *o*-aminoacetophenone (**31**), 2-phenylethanol (**32**), and phenylacetone (**33**). 6-Methylhept-5-en-2-one (**34**) was detected in all strains, while its saturated analog 6-methylheptan-2-one (**35**) was only emitted by *C. baekdonensis* and geranylacetone (**36**) only by the three productive species *C. marinus*, *C. neptunius*, and *C. manganoxidans*. Compounds **34** and **36** have been described as non-enzymatic degradation products arising from the side chain in menaquinones [58]. Sulfur-containing compounds included dimethyl trisulfide (**37**), released by all six species, *S*-methyl methanethiosulfonate (**38**), 2-acetylthiazole (**39**), and benzothiazole (**40**), the latter also in the extracts from all six strains. In addition, the extracts from the three species *C. marinus*, *C. neptunius* and *C. baekdonensis* contained an additional volatile (**41**) whose mass spectrum (Figure 3A) was not included in our libraries. Furthermore, ethyl 3-(methylsulfanyl)acrylate (**42**) was found in *C. marinus* and *C. manganoxidans*, but the measured retention index (*I* = 1177) did not allow to distinguish between the *E* and the *Z* isomer for which retention indices of *I* = 1144 (*E*) and *I* = 1158 (*Z*) were reported [53]. Therefore, for an unambiguous structural assignment for compounds **41** and **42** the synthesis of reference compounds was required.

### Synthesis of reference compounds

**Scheme 2:**
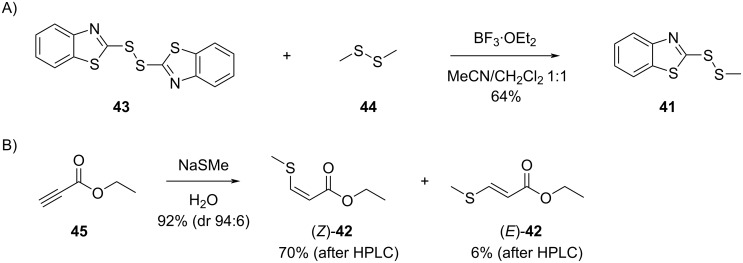
Synthesis of sulfur-containing compounds detected in the *Celeribacter* headspace extracts. A) Synthesis of 2-(methyldisulfanyl)benzothiazole (**41**) and B) synthesis of ethyl (*Z*)- and (*E*)-3-(methylsulfanyl)acrylate (**42**).

The mass spectrum of the component **41** showed strong similarities to the library mass spectrum of 2-mercaptobenzothiazole that has a molecular weight of 167 Da. The isotope pattern of the molecular ion at *m*/*z* = 213 indicated the presence of three sulfur atoms. The strong base peak at *m*/*z* = 167 in the mass spectrum of **41** suggested a benzothiazol-2-ylsulfanyl moiety, while the mass difference to the molecular ion pointed to the connection to a methylsulfanyl group. Taken together, this analysis resulted in the structural proposal of 2-(methyldisulfanyl)benzothiazole for **41**. For the structural verification a synthesis was performed by a BF_3_·OEt_2_-catalyzed reaction of bis(benzothiazol-2-yl)disulfane with dimethyl disulfide, giving access to **41** with a yield of 64% (Scheme 2). The synthetic compound **41** showed an identical mass spectrum and retention index compared to the volatile in the *Celeribacter* extracts. The *Z* and *E* stereoisomers of **42** were obtained by the Michael addition of NaSMe to ethyl propiolate (**45**), yielding a mixture of stereoisomers inseparable by silica gel column chromatography (92%). The major stereoisomer was found to be (*Z*)-**42** (dr 94:6), whose preferred formation may be a result of a chalcogen–chalcogen interaction between the sulfur and an ester oxygen. This phenomenon was first described in supramolecular structures by Gleiter [59] and later also used to explain the outcome of organocatalytic reactions [60]. The pure stereoisomers of **42** were isolated by preparative HPLC, for which the best separation was achieved using a YMC ChiralART Cellulose-SC column. This yielded 70% of (*Z*)-**42** and 6% of (*E*)-**42**, and their analysis by GC–MS showed retention indices of *I* = 1177 for (*E*)-**42** and *I* = 1200 for (*Z*)-**42**, revealing that the compound in the headspace extracts of *C. marinus* DSM 100036^T^ and *C. manganoxidans* DSM 27541^T^ was identical to (*E*)-**42**.

### Feeding experiments with isotopically labeled precursors

The biosynthesis of sulfur volatiles in *C. marinus* was investigated in a series of feeding experiments with isotopically labeled precursors. Feeding of (*methyl*-^2^H_3_)methionine resulted in the efficient incorporation of labeling into **37** (79% incorporation rate, Figure S1B in Supporting Information File 1), **38** (78%, Figure S1F in Supporting Information File 1) and the *S*-methyl group of **41** (84%), as indicated by a shift of the molecular ion from *m*/*z* = 213 to 216 (Figure 3B, deuterated compounds can be separated from their non-deuterated analogs by gas chromatography [61]). The base peak appears at *m*/*z* = 168, demonstrating its formation with participation of one deuterium from the *S*-methyl group. Analogous results were obtained by feeding of (*methyl*-^13^C)methionine, showing incorporation into **37** (74%, Figure S1C in Supporting Information File 1), **38** (71%, Figure S1G in Supporting Information), and the MeS group of **41** (71%, Figure 3C; the signal at *m*/*z* = 213 represents unlabeled **41** that, in contrast to a deuterated compound, cannot be separated from ^13^C-labeled **41** by gas chromatography). Furthermore, feeding of [^34^S]DMSP gave an incorporation into the MeS groups of **37** (50%, Figure S1D), into both sulfur atoms of **38** (47%, Figure S1H in Supporting Information File 1), but only into one sulfur atom of **41** (46%), as indicated by the molecular ion at *m*/*z* = 215, while no signals at *m*/*z* = 217 and 219 were visible that would account for the incorporation of labeling into two or three of the sulfur atoms in **41** (Figure 3D; also here the signal at *m*/*z* = 213 represents inseparable unlabeled **41**). In this experiment, the base peak did not change which allowed the localization of labeling specifically in the MeS group of **41**.

The fact that no incorporation was observed for the other two sulfur atoms of **41** prompted us to further investigate the biosynthetic origin of the benzothiazol-2-ylsulfanyl portion of **41** to establish its natural origin. Several feeding experiments with central primary metabolites including (^13^C_6_)glucose, (^13^C_5_)ribose and (*indole*-^2^H_5_)tryptophan were performed, but none of these experiments resulted in a detectable incorporation of labeling. Conclusively, a non-biological origin of this part of the molecule seems likely, which may also explain why the detection of **41** in *Celeribacter* was not always reproducible. Notably, 2-mercaptobenzothiazole is used in the sulfur vulcanization of rubber and could react spontaneously with MeSH of bacterial origin in the presence of oxygen to form **41**, giving a reasonable explanation for its formation.

Also none of the feeding experiments with the various labeled precursors resulted in an incorporation of labeling into the sulfur volatiles **39**, **40**, and **42**, which also questioned their natural origin. This finding is rather surprising for **42**, especially regarding the feeding experiment with (^34^S)DMSP, because its formation would be explainable by a DMSP degradation through the demethylation pathway, for which all relevant enzymes are encoded in the six *Celeribacter* strains (only a DmdA homolog is missing in *C. indicus*, Table S1 in Supporting Information File 1), and e.g., transesterification of the DmdC product with EtOH (Scheme 1A). Compound **42** is not a widespread sulfur volatile, but has been reported before from pineapples [53], pears [62], passion fruits [63], and apples [64].

## Conclusion

Six marine *Celeribacter* strains were investigated for their volatiles, leading to the identification of 42 compounds from different classes, including several sulfur volatiles. However, feeding experiments with isotopically labeled precursors suggested that only the widespread compounds dimethyl trisulfide (**37**) and *S*-methyl methanethiosulfonate (**38**) are of natural origin, while no labeling from any of the fed precursors was incorporated into 2-acetylthiazole (**39**), benzothiazole (**40**), and ethyl (*E*)-3-(methylsulfanyl)acrylate (**42**), thus questioning their natural source from *Celeribacter*. These results demonstrate that the six *Celeribacter* strains are able to degrade methionine and DMSP with formation of MeSH as a source for the likely non-enzymatic oxidation in the presence of air to **37** and **38**, opening possibilities for future studies on methionine and DMSP degrading enzymes and pathways in *Celeribacter*. Our study also shows that the results from trace compound analyses must be taken with care and contaminations from other sources must always be taken into consideration. For the unusual compound 2-(methyldisulfanyl)benzothiazole (**41**) the incorporation of labeling was observed only into the MeS group, while the benzothiazol-2-ylsulfanyl portion is likely of anthropogenic origin from the rubber vulcanization agent 2-mercaptobenzothiazole that reacts with MeSH from the bacterial metabolism.

## Experimental

### Strains, culture conditions, and feeding experiments

All six *Celeribacter* type strains were cultivated at 28 °C on marine broth agar plates. In case of feeding experiments, the isotopically labeled compound (1 mM) was added to the agar medium before inoculation.

### Collection of volatiles

The volatiles emitted by *Celeribacter* spp. agar plate cultures were collected on charcoal filters (Chromtech, Idstein, Germany, precision charcoal filters charged with 5 mg of charcoal) by use of a closed-loop stripping apparatus as developed by Grob and Zürcher [37]. After a collection time of 24 h the charcoal was extracted with CH_2_Cl_2_ (50 μL) and the extract was analyzed by GC–MS.

### GC–MS

GC–MS analyses were carried out through a 7890B GC – 5977A MD system (Agilent, Santa Clara, CA, USA). The GC was equipped with a HP5-MS fused silica capillary column (30 m, 0.25 mm i.d., 0.50 μm film) and operated with the settings 1) inlet pressure: 77.1 kPa, He flow: 23.3 mL min^−1^, 2) injection volume: 2 μL, 3) splitless injection, 4) temperature program: 5 min isothermic at 50 °C, then increasing with 5 °C min^−1^ to 320 °C, and 5) He carrier gas flow: 1.2 mL min^−1^. The parameters of the MS were 1) transfer line temperature: 250 °C, 2) ion source temperature: 230 °C, 3) quadrupole temperature: 150 °C, and 4) electron energy: 70 eV. Retention indices were calculated from retention times in comparison to those of a homologous series of *n*-alkanes (C_7_–C_40_).

### General synthetic and analytical methods

Reactions were carried out in oven-dried flasks under Ar atmosphere and using distilled and dried solvents. Chemicals were obtained from Sigma-Aldrich (St. Louis, USA). Column chromatography was performed on silica gel (0.04–0.06 nm) purchased from Acros Organics (Geel, Belgium) with distilled solvents. NMR spectroscopy was performed on a Bruker (Billerica, USA) Avance III HD Ascend (500 MHz) spectrometer. Solvent peaks were used for referencing (^1^H NMR: CDCl_3_ residual proton signal δ = 7.26 ppm, ^13^C NMR: CDCl_3_ δ = 77.16 ppm) [65]. Multiplicities are indicated by s (singlet) and d (doublet), coupling constants *J* are given in Hz. IR spectra were recorded on a Bruker α spectrometer equipped with a diamond-ATR probe, and qualitative signal intensities are reported by w (weak), m (medium), and s (strong). HPLC purification of compound **42** was performed on an Azura HPLC system (Knauer, Berlin, Germany) equipped with a UV–vis detector MWL 2.1L (deuterium lamp, 190–700 nm) and a YMC ChiralART Cellulose-SC column (5 μm; 250 × 20 mm) with a guard column of the same type (30 × 20 mm). The elution was performed with hexane/propanol 60:40 (isocratic) at a flow rate of 10 mL min^−1^ (36 bar). The UV–vis absorption was monitored at 275 nm.

### Synthesis of 2-(methyldisulfanyl)benzothiazole (**41**)

1,2-Bis(benzothiazol-2-yl)disulfane (**43**, 1.00 g, 3.00 mmol, 1 equiv) and dimethyl sulfide (**44**, 0.28 g, 3.00 mmol, 1 equiv) were dissolved in dry CH_3_NO_2_ (10 mL) and dry CH_2_Cl_2_ (10 mL). The solution was cooled to 0 °C and then treated with BF_3_·Et_2_O (43 mg, 0.3 mmol, 0.1 equiv). After stirring at 0 °C for 3 hours and at room temperature overnight, the reaction was quenched by the addition of water (10 mL) and extracted with ethyl acetate (3 × 50 mL). The combined extracts were dried with MgSO_4_ and concentrated. The residue was purified by column chromatography (cyclohexane/ethyl acetate 1:1) to give **41** as a colorless solid (0.82 g, 3.85 mmol, 64%). *R*_f_ 0.60 (cyclohexane/ethyl acetate 5:1; TLC visualized with UV illumination at 366 nm); GC (HP-5MS): *I* = 1854; IR (diamond-ATR) ν̃: 3060 (s), 2916 (s), 1425 (w), 1310 (s), 1236 (s), 1005 (w), 756 (w), 431 (s) cm^−1^; ^1^H NMR (500 MHz, CDCl_3_, 298 K) δ 7.88 (ddd, *J* = 8.1, 1.2, 0.7 Hz, 1H, CH), 7.87 (ddd, *J* = 7.9, 1.2, 0.6 Hz, 1H, CH), 7.43 (ddd, *J* = 8.3, 7.3, 1.2 Hz, 1H, CH), 7.33 (ddd, *J* = 8.2, 7.2, 1.2 Hz, 1H, CH), 2.67 (s, 3H, CH_3_) ppm; ^13^C NMR (125 MHz, CDCl_3_, 298 K) δ 172.50 (C), 155.17 (C), 135.90 (C), 126.37 (CH), 124.70 (CH), 122.24 (CH), 121.27 (CH), 23.62 (CH_3_) ppm.

### Synthesis of ethyl (*Z*)-3-(methylsulfanyl)acrylate ((*Z*)-**42**) and ethyl (*E*)-3-(methylsulfanyl)acrylate ((*E*)-**42**)

Ethyl propiolate (**45**, 70 mg, 0.71 mmol, 1 equiv) was dissolved in distilled water (5 mL) followed by the addition of sodium methanethiolate (50 mg, 0.71 mmol, 1 equiv). The solution was stirred for 30 minutes at room temperature. Water (5 mL) was added and the product was extracted with ethyl acetate (3 × 10 mL). The combined extracts were dried over MgSO_4_ and concentrated to afford the crude product. Purification by column chromatography (cyclohexane/ethyl acetate 99:1) gave a mixture of stereoisomers (*Z*)-**42** and (*E*)-**42** as pale yellow oil (96 mg, 0.65 mmol, 92%, dr 94:6 by ^1^H NMR). The product mixture was separated by preparative HPLC to give pure (*Z*)-**42** (73 mg, 0.50 mmol, 70%) and (*E*)-**42** (6 mg, 0.04 mmol, 6%).

**(*Z*)-42.** *R*_f_ 0.74 (cyclohexane/ethyl acetate 1:1); GC (HP-5MS): *I* = 1200; IR (diamond-ATR) ν̃: 2982 (w), 2927 (w),1695 (m), 1569 (m), 1434 (w), 1374 (w), 1300 (w), 1266 (w), 1213 (m), 1166 (s), 1095 (w), 1033 (w), 986 (w), 961 (w), 800 (w), 727 (w), 687 (w) cm^−1^; ^1^H NMR (700 MHz, CDCl_3_, 298 K) δ 7.04 (d, *J* = 10.14 Hz, 1H, CH), 5.83 (d, *J* = 10.14 Hz, 1H, CH), 4.20 (q, *J* = 7.15 Hz, 2H, CH_2_), 2.39 (s, 3H, CH_3_), 1.29 (t, *J* = 7.17 Hz, 3H, CH_3_) ppm; ^13^C NMR (175 MHz, CDCl_3_, 298 K) δ 166.75 (C), 151.84 (CH), 113.18 (CH), 60.17 (CH_2_), 19.28 (CH_3_), 14.44 (CH_3_) ppm.

**(*E*)-42.** *R*_f_ 0.76 (cyclohexane/ethyl acetate 1:1); GC (HP-5MS): *I* = 1177; IR (diamond-ATR) ν̃: 2980 (w), 2925 (w), 1701 (s), 1578 (s), 1444 (w), 1366 (w), 1322 (w), 1297 (m), 1251 (s), 1161 (s), 1095 (w), 1037 (m), 945 (m), 886 (w), 832 (w), 799 (w), 702 (w) cm^−1^; ^1^H NMR (700 MHz, CDCl_3_, 298 K) δ 7.76 (d, *J* = 14.93 Hz, 1H, CH), 5.68 (d, *J* = 14.90 Hz, 1H, CH), 4.21 (q, *J* = 7.14 Hz, 2H, CH_2_), 2.35 (s, 3H, CH_3_), 1.31 (t, *J* = 7.13 Hz, 3H, CH_3_) ppm; ^13^C NMR (175 MHz, CDCl_3_, 297 K) δ 165.59 (C), 147.21 (CH),113.56 (CH), 60.55 (CH_2_), 27.26 (CH_3_), 14.67 (CH_3_) ppm.

## Supporting Information

File 1 DMSP demethylation pathway in *Celeribacter* spp. and copies of spectra.

## References

[1] Giovannoni S J, Stingl U (2005). Nature.

[2] González J M, Moran M A (1997). Appl Environ Microbiol.

[3] Selje N, Simon M, Brinkhoff T (2004). Nature.

[4] Brinkhoff T, Giebel H-A, Simon M (2008). Arch Microbiol.

[5] Lee O O, Tsoi M M Y, Li X, Wong P-K, Qian P-Y (2007). Int J Syst Evol Microbiol.

[6] Ruiz-Ponte C, Cilia V, Lambert C, Nicolas J L (1998). Int J Syst Bacteriol.

[7] Shiba T (1991). Syst Appl Microbiol.

[8] Biebl H, Allgaier M, Tindall B J, Koblizek M, Lünsdorf H, Pukall R, Wagner-Döbler I (2005). Int J Syst Evol Microbiol.

[9] Lafay B, Ruimy R, Rausch de Traubenberg C, Breittmayer V, Gauthier M J, Christen R (1995). Int J Syst Bacteriol.

[10] González J M, Simó R, Massana R, Covert J S, Casamayor E O, Pedrós-Alió C, Moran M A (2000). Appl Environ Microbiol.

[11] Dickschat J S, Rabe P, Citron C A (2015). Org Biomol Chem.

[12] Reisch C R, Stoudemayer M J, Varaljay V A, Amster I J, Moran M A, Whitman W B (2011). Nature.

[13] Todd J D, Rogers R, Li Y G, Wexler M, Bond P L, Sun L, Curson A R J, Malin G, Steinke M, Johnston A W B (2007). Science.

[14] Todd J D, Kirkwood M, Newton-Payne S, Johnston A W B (2012). ISME J.

[15] Kirkwood M, Le Brun N E, Todd J D, Johnston A W B (2010). Microbiology (London, U K).

[16] Todd J D, Curson A R J, Kirkwood M, Sullivan M J, Green R T, Johnston A W B (2011). Environ Microbiol.

[17] Curson A R J, Rogers R, Todd J D, Brearley C A, Johnston A W B (2008). Environ Microbiol.

[18] Curson A R J, Sullivan M J, Todd J D, Johnston A W B (2011). ISME J.

[19] Sun J, Todd J D, Thrash J C, Qian Y, Qian M C, Temperton B, Guo J, Fowler E K, Aldrich J T, Nicora C D (2016). Nat Microbiol.

[20] Lovelock J E, Maggs R J, Rasmussen R A (1972). Nature.

[21] Charlson R J, Lovelock J E, Andreae M O, Warren S G (1987). Nature.

[22] Dickschat J S, Zell C, Brock N L (2010). ChemBioChem.

[23] Brock N L, Citron C A, Zell C, Berger M, Wagner-Döbler I, Petersen J, Brinkhoff T, Simon M, Dickschat J S (2013). Beilstein J Org Chem.

[24] Brock N L, Menke M, Klapschinski T A, Dickschat J S (2014). Org Biomol Chem.

[25] Celik E, Maczka M, Bergen N, Brinkhoff T, Schulz S, Dickschat J S (2017). Org Biomol Chem.

[26] Durham B P, Sharma S, Luo H, Smith C B, Amin S A, Bender S J, Dearth S P, Van Mooy B A S, Campagna S R, Kujawinski E B (2015). Proc Natl Acad Sci U S A.

[27] Mönnich J, Tebben J, Bergemann J, Case R, Wohlrab S, Harder T (2020). ISME J.

[28] Bruhn J B, Nielsen K F, Hjelm M, Hansen M, Bresciani J, Schulz S, Gram L (2005). Appl Environ Microbiol.

[29] Greer E M, Aebisher D, Greer A, Bentley R (2008). J Org Chem.

[30] Kintaka K, Ono H, Tsubotani S, Harada S, Okazaki H (1984). J Antibiot.

[31] Geng H, Bruhn J B, Nielsen K F, Gram L, Belas R (2008). Appl Environ Microbiol.

[32] Berger M, Brock N L, Liesegang H, Dogs M, Preuth I, Simon M, Dickschat J S, Brinkhoff T (2012). Appl Environ Microbiol.

[33] Brock N L, Nikolay A, Dickschat J S (2014). Chem Commun.

[34] Beyersmann P G, Tomasch J, Son K, Stocker R, Göker M, Wagner-Döbler I, Simon M, Brinkhoff T (2017). Sci Rep.

[35] Thiel V, Brinkhoff T, Dickschat J S, Wickel S, Grunenberg J, Wagner-Döbler I, Simon M, Schulz S (2010). Org Biomol Chem.

[36] Seyedsayamdost M R, Case R J, Kolter R, Clardy J (2011). Nat Chem.

[37] Grob K, Zürcher F (1976). J Chromatogr.

[38] Kováts E (1958). Helv Chim Acta.

[39] Elmore J S, Mottram D S, Enser M, Wood J D (2000). Meat Sci.

[40] Dickschat J S, Wickel S, Bolten C J, Nawrath T, Schulz S, Wittmann C (2010). Eur J Org Chem.

[41] Cerny C, Guntz-Dubini R (2006). J Agric Food Chem.

[42] Spadone J-C, Takeoka G, Liardon R (1990). J Agric Food Chem.

[43] Lee S-R, Macku C, Shibamoto T (1991). J Agric Food Chem.

[44] Pino J A, Mesa J, Muñoz Y, Martí M P, Marbot R (2005). J Agric Food Chem.

[45] Adams R P (2009). Identification of Essential Oil Components by Gas Chromatography/Mass Spectrometry.

[46] Ansorena D, Gimeno O, Astiasarán I, Bello J (2001). Food Res Int.

[47] Citron C A, Rabe P, Dickschat J S (2012). J Nat Prod.

[48] Owens J D, Allagheny N, Kipping G, Ames J M (1997). J Sci Food Agric.

[49] Cha Y J, Cadwallader K R (1998). J Agric Food Chem.

[50] Wickel S M, Citron C A, Dickschat J S (2013). Eur J Org Chem.

[51] Kubec R, Drhová V, Velíšek J (1998). J Agric Food Chem.

[52] Ferhat M A, Tigrine-Kordjani N, Chemat S, Meklati B Y, Chemat F (2007). Chromatographia.

[53] Takeoka G R, Buttery R G, Teranishi R, Flath R A, Güntert M (1991). J Agric Food Chem.

[54] Nawrath T, Mgode G F, Weetjens B, Kaufmann S H E, Schulz S (2012). Beilstein J Org Chem.

[55] Citron C A, Barra L, Wink J, Dickschat J S (2015). Org Biomol Chem.

[56] Schulz S, Dickschat J S (2007). Nat Prod Rep.

[57] Dickschat J S (2017). Nat Prod Rep.

[58] Ueda D, Matsugane S, Okamoto W, Hashimoto M, Sato T (2018). Angew Chem, Int Ed.

[59] Werz D B, Staeb T H, Benisch C, Rausch B J, Rominger F, Gleiter R (2002). Org Lett.

[60] Leverett C A, Purohit V C, Romo D (2010). Angew Chem, Int Ed.

[61] Dickschat J S (2014). Nat Prod Rep.

[62] Takeoka G R, Buttery R G, Flath R A (1992). J Agric Food Chem.

[63] Werkhoff P, Güntert M, Krammer G, Sommer H, Kaulen J (1998). J Agric Food Chem.

[64] Ferreira L, Perestrelo R, Caldeira M, Câmara J S (2009). J Sep Sci.

[65] Fulmer G R, Miller A J M, Sherden N H, Gottlieb H E, Nudelman A, Stoltz B M, Bercaw J E, Goldberg K I (2010). Organometallics.

