# Supplementary material for: Identification of volatiles from six marine Celeribacter strains
Source: Beilstein J Org Chem. 2021 Feb 11;17:420–30. doi: 10.3762/bjoc.17.38 (PMC7884881; doi:10.3762/bjoc.17.38)
Supplement: File 1 — DMSP demethylation pathway in Celeribacter spp. and copies of spectra. [file Beilstein_J_Org_Chem-17-420-s001.pdf]

BEILSTEIN JOURNAL OF ORGANIC CHEMISTRY

Supporting Information

for

Identification of volatiles from six marine *Celeribacter* strains

Anuj Kumar Chhalodia, Jan Rinkel, Dorota Konvalinkova, Jörn Petersen
and Jeroen S. Dickschat

Beilstein J. Org. Chem. **2021,** *17,* 420–430. doi:10.3762/bjoc.17.38

DMSP demethylation pathway in *Celeribacter* spp. and copies of spectra

License and Terms: This is a supporting information file under the terms of the Creative Commons Attribution License (https://creativecommons.org/licenses/by/4.0). Please note that the reuse, redistribution and reproduction in particular requires that the author(s) and source are credited and that individual graphics may be subject to special legal provisions.
The license is subject to the *Beilstein Journal of Organic Chemistry* terms and conditions: (https://www.beilstein-journals.org/bjoc/terms)

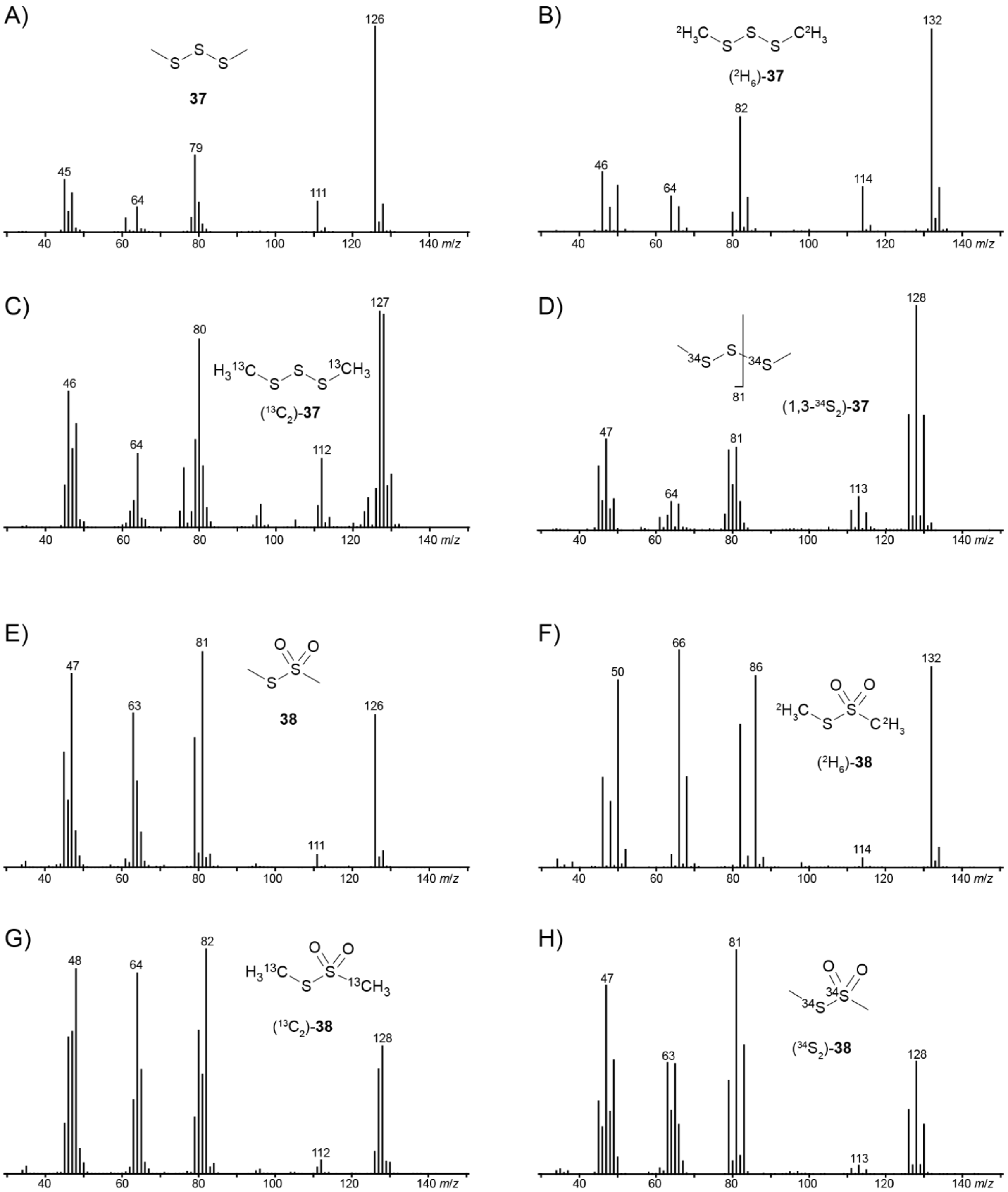

Figure S1: EI mass spectra of A) unlabeled dimethyl trisulfide (**37**) and of labeled **37** after feeding of B) [*methyl*-$^2H_3$]methionine, C) [*methyl*-^{13}C]methionine and D) [^{34}S]DMSP, E) unlabeled S-methyl methanethiosulfonate (**38**) and of labeled **38** after feeding of F) [*methyl*-$^2H_3$]methionine, G) [*methyl*-^{13}C]methionine, and H) [^{34}S]DMSP. The structures of labeled compounds show the isotopomers of maximum incorporation of labeling.

Table S1: The DMSP demethylation pathway in *Celeribacter* spp.

Strain	DmdA[a]	DmdB[a]	DmdC[a]	DmdD[a]
C. marinus DSM 100036[T]	WP_062220065 (21%)	WP_062217242 (22%)	WP_062219918 (38%)	WP_062217896 (30%)
C. neptunius DSM 26471[T]	WP_090061255 (26%)[b]	WP_090061186 (25%)	WP_090062142 (40%)	WP_090062168 (43%)
C. manganoxidans DSM 27541[T]	WP_097373155 (21%)[b]	WP_097373111 (31%)	WP_097372585 (41%)	WP_088662863 (30%)
C. baekdonensis DSM 27375[T]	WP_074644298 (28%)[b]	WP_107720993 (32%)	WP_107720980 (46%)	WP_074641224 (43%)
C. halophilus DSM 26270[T]	WP_066599093 (25%)[b]	WP_066604139 (26%)	WP_066603281 (39%)	WP_066601109 (31%)
C. indicus DSM 27257[T]	–	WP_139267207 (27%)	WP_043868654 (40%)	WP_043869106 (42%)

[a]NCBI accession numbers and % identity of the closest homologs of the functionally characterized enzymes DmdABCD from *Ruegeria pomeroyi* DSS-3 obtained by BLAST search; [b]identity by comparison to C-terminal domain.

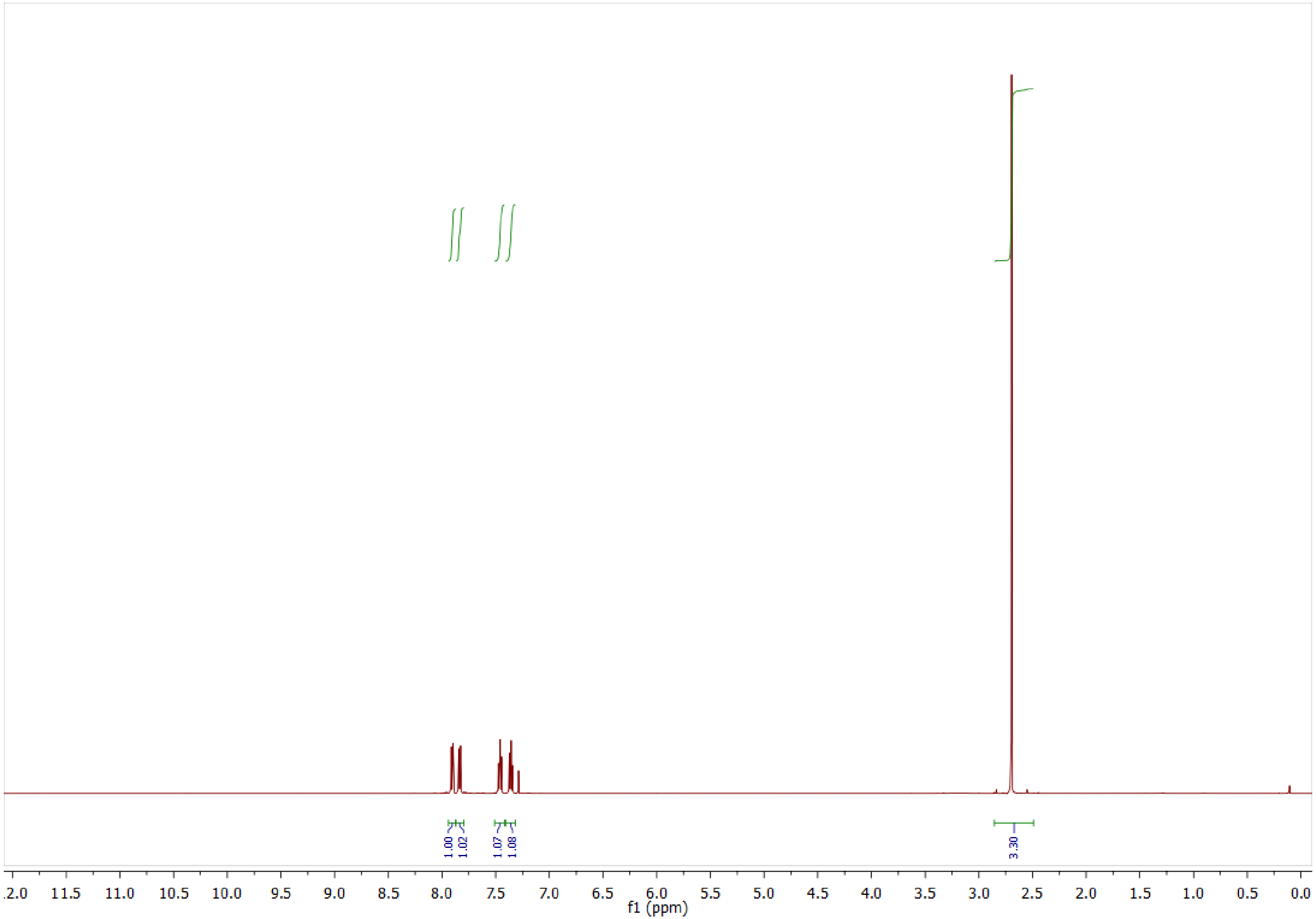

Figure S2: ^{1}H NMR spectrum ($CDCl_3$, 500 MHz) of **41**.

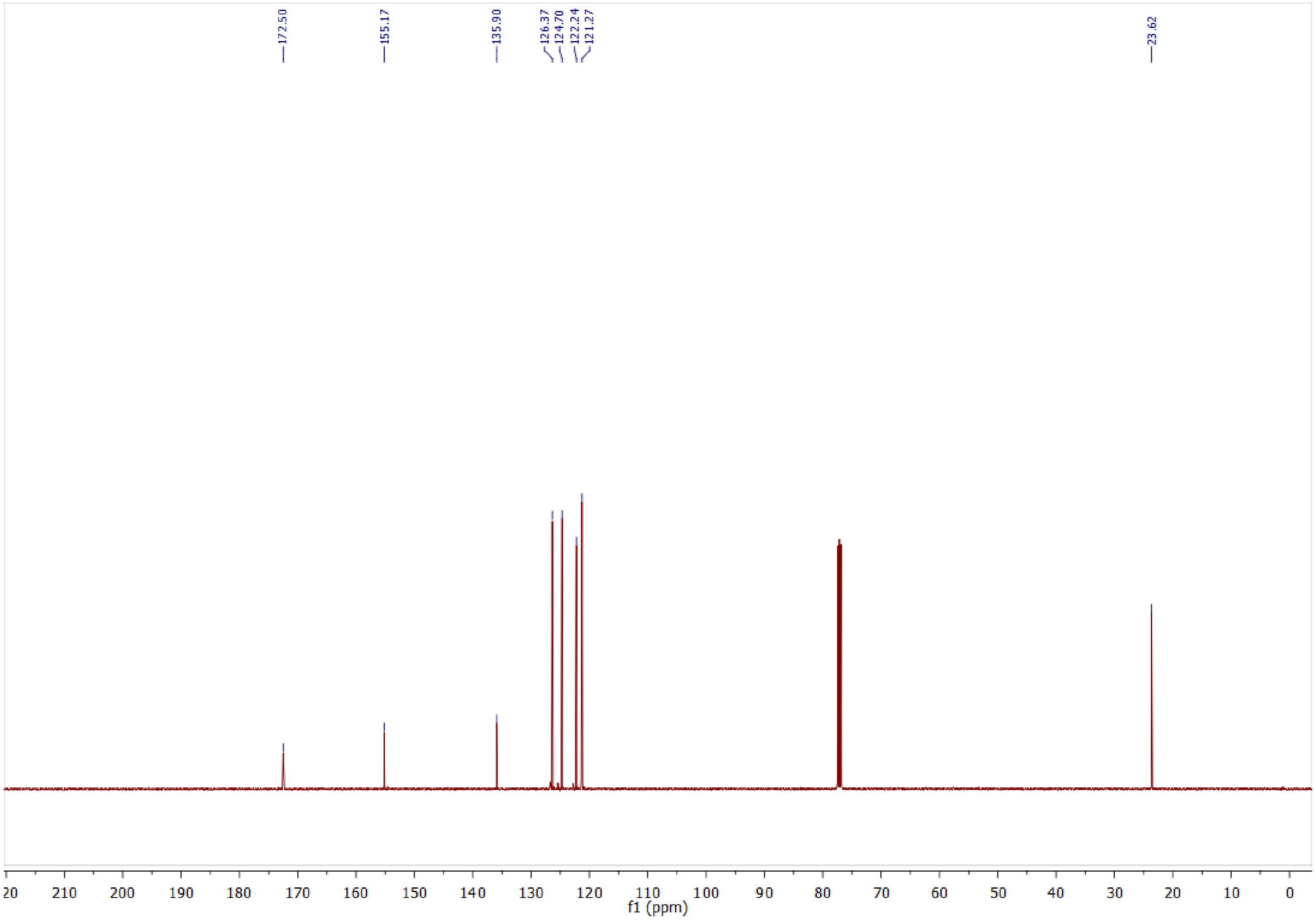

Figure S3: ^{13}C NMR spectrum ($CDCl_3$, 125 MHz) of **41**.

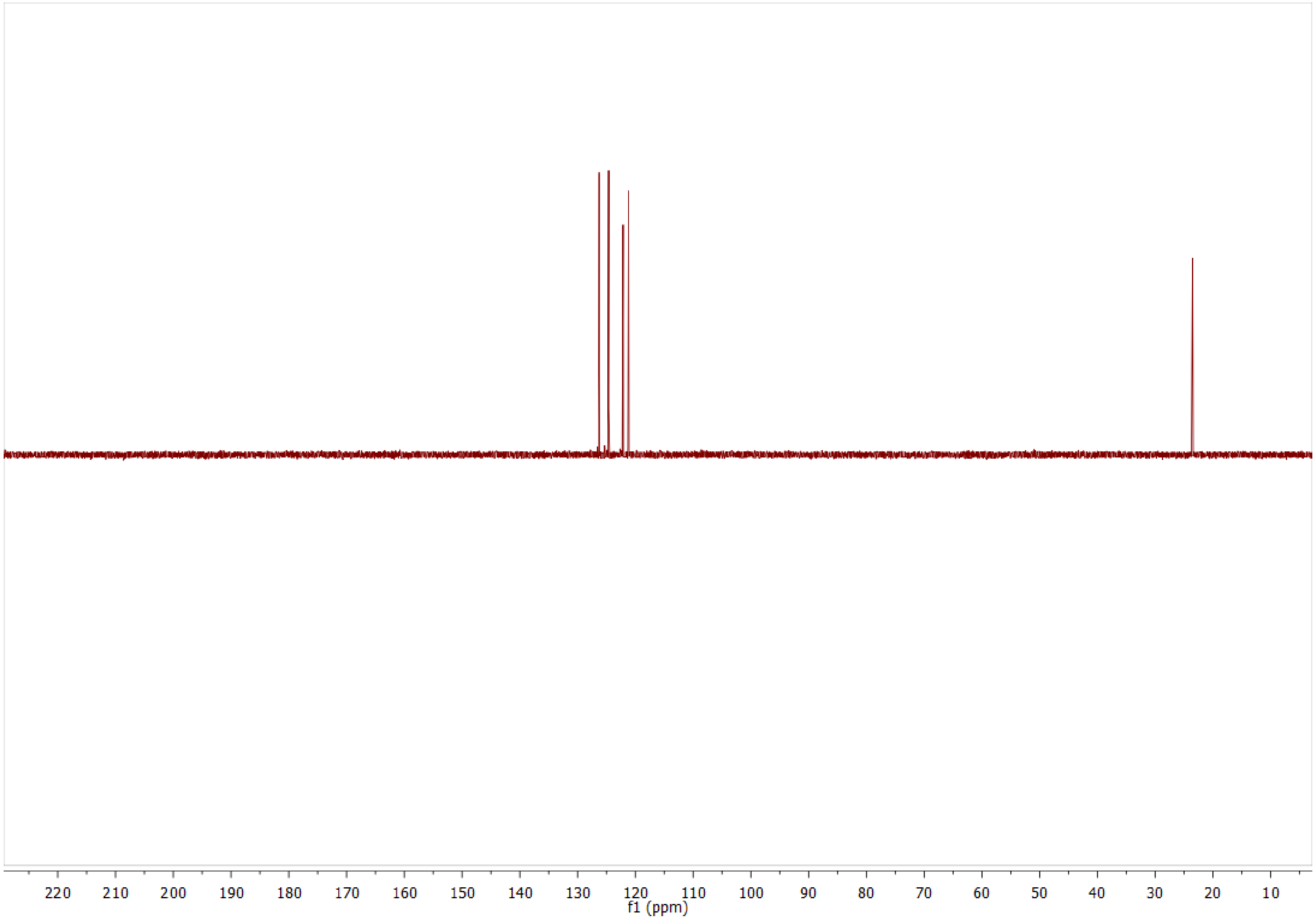

Figure S4: ^{13}C-DEPT spectrum ($CDCl_3$, 125 MHz) of **41**.

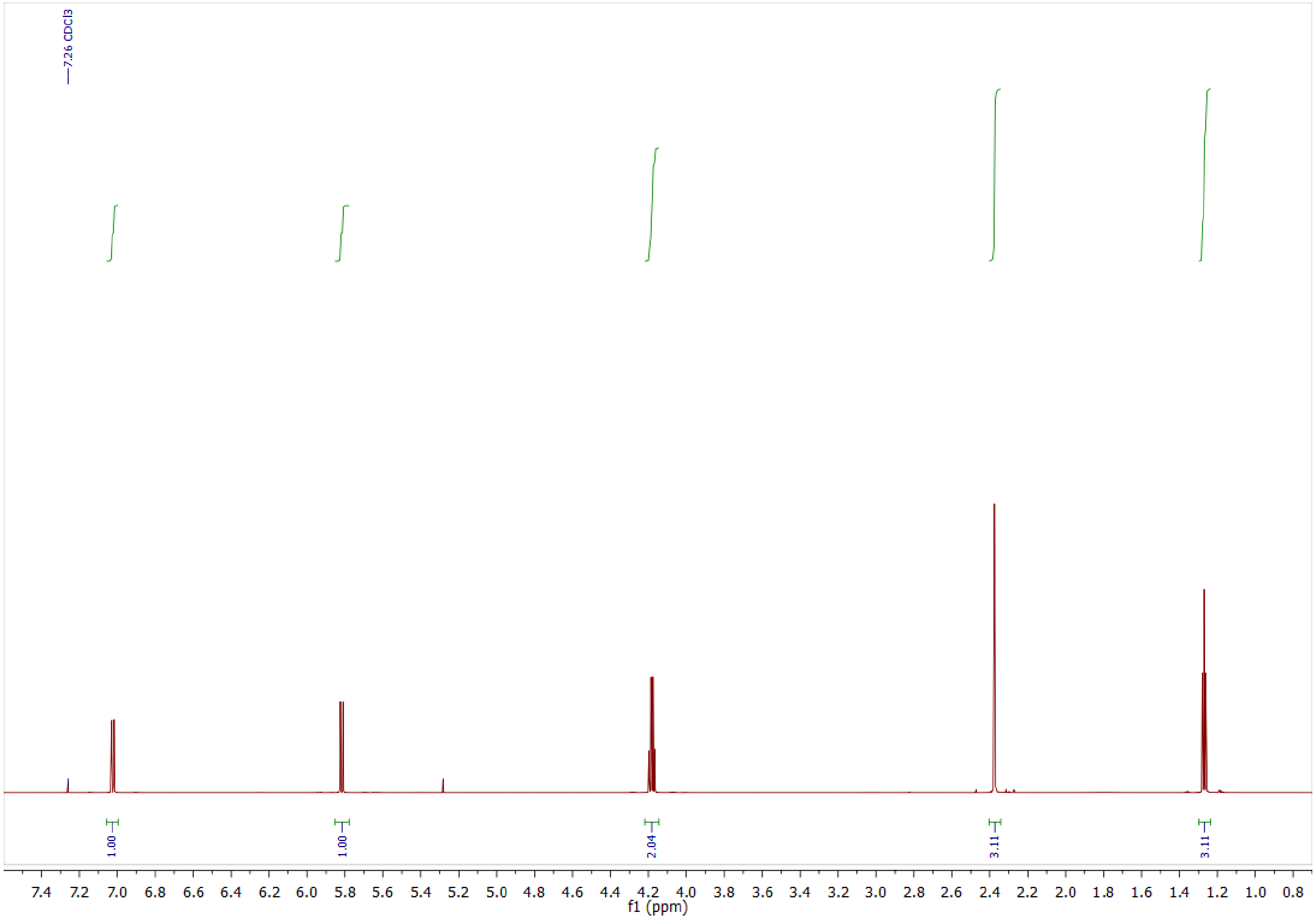

Figure S5: ^{1}H NMR spectrum ($CDCl_3$, 700 MHz) of (*Z*)-**42**.

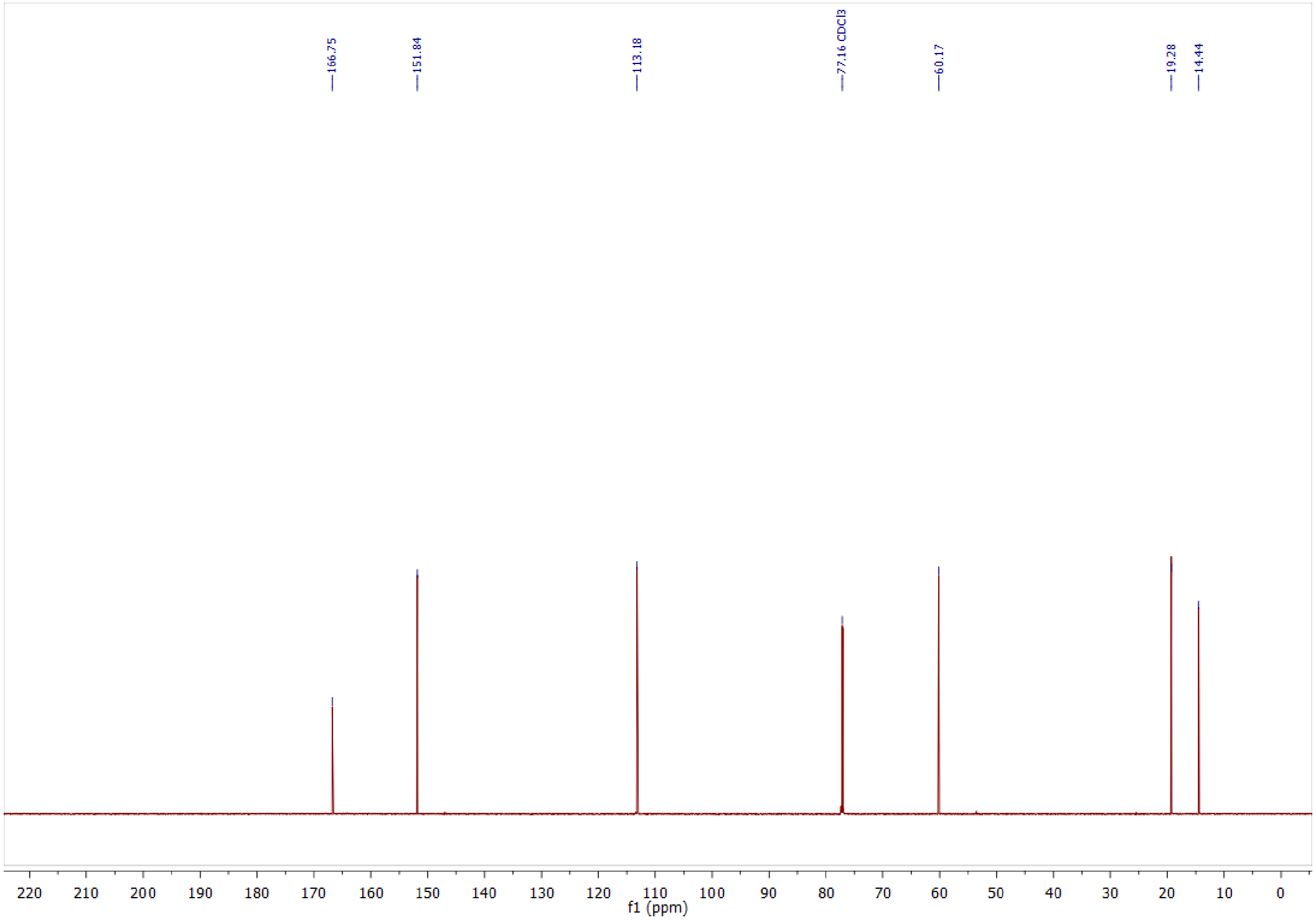

Figure S6: ^{13}C NMR spectrum ($CDCl_3$, 175 MHz) of (*Z*)-**42**.

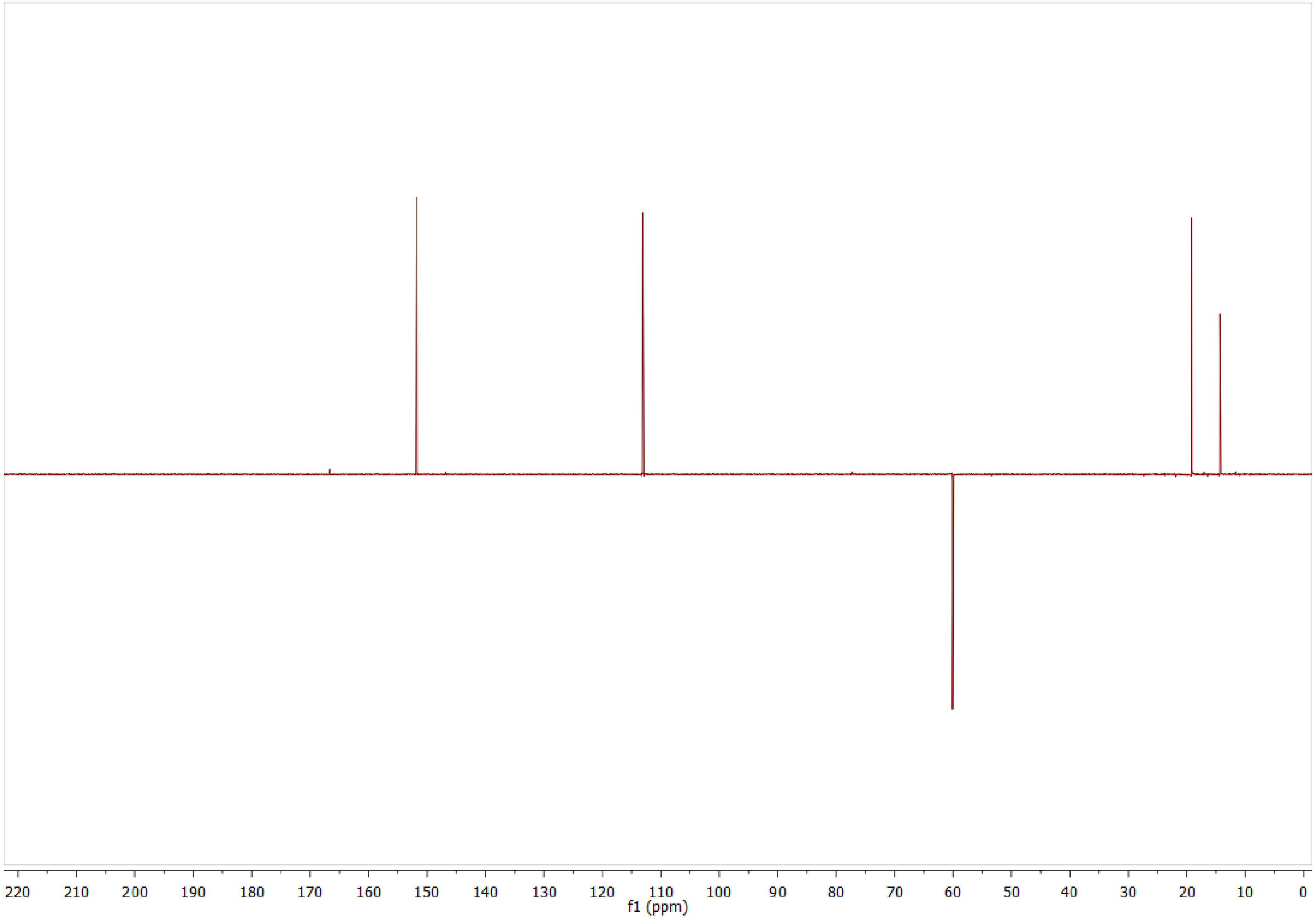

Figure S7: ^{13}C-DEPT spectrum (D_2O, 175 MHz) of (*Z*)-**42**.

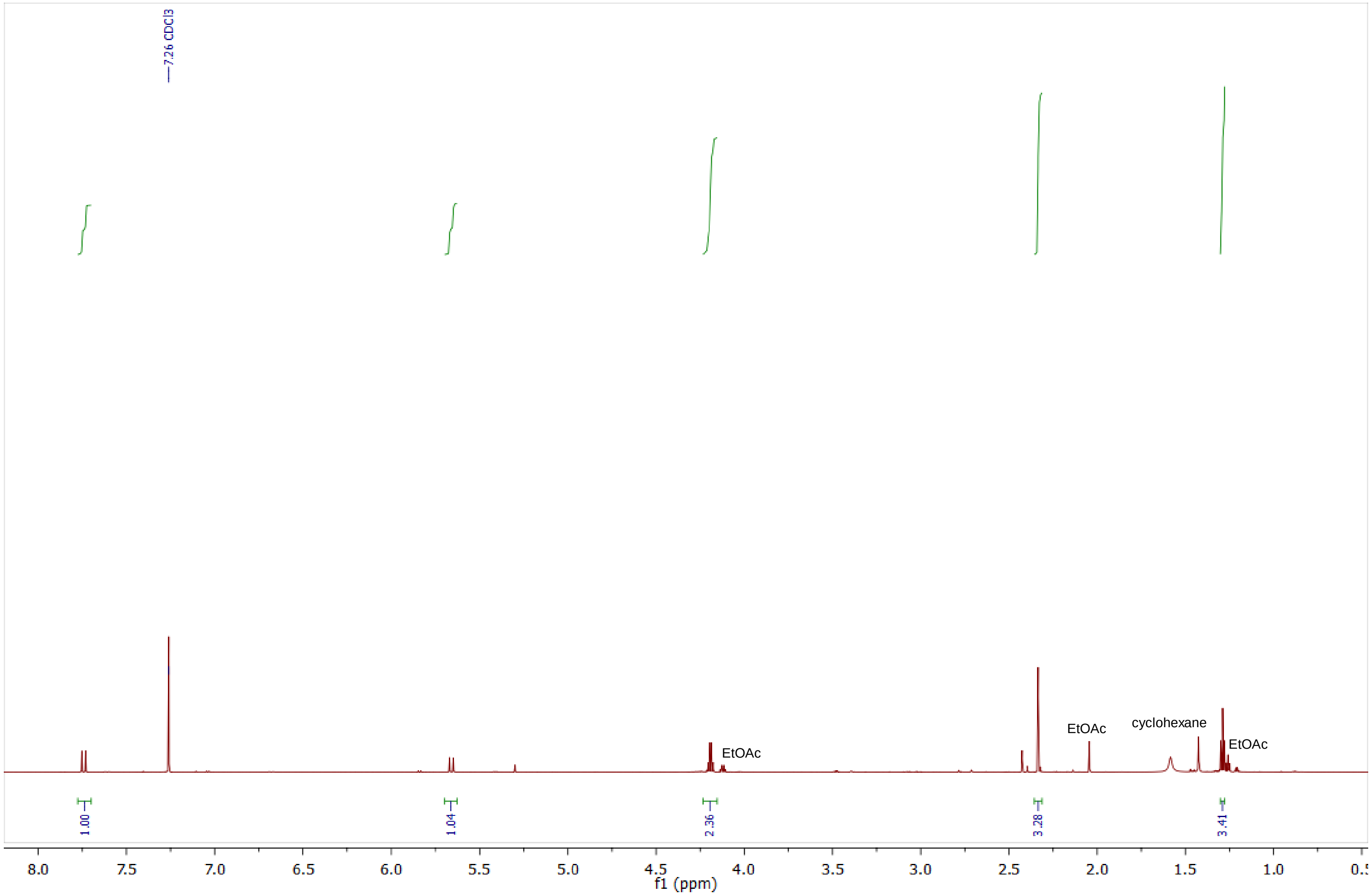

Figure S8: ^{1}H NMR spectrum ($CDCl_3$, 700 MHz) of (*E*)-**42**. Minor impurities originate from solvents which were not rigorously removed, because the compound was obtained in small amounts and is volatile.

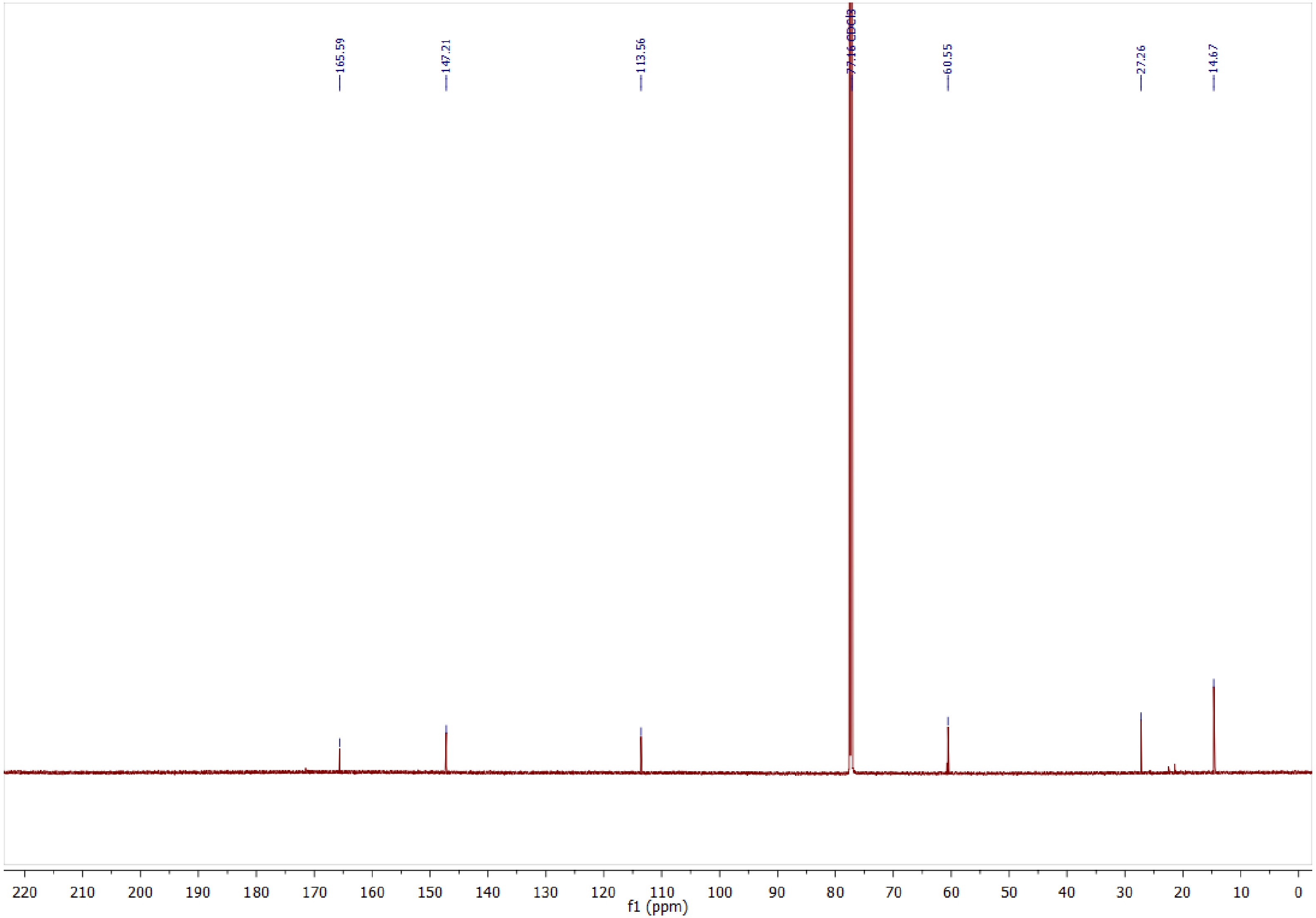

Figure S9: ^{13}C NMR spectrum ($CDCl_3$, 175 MHz) of (*E*)-**42**.

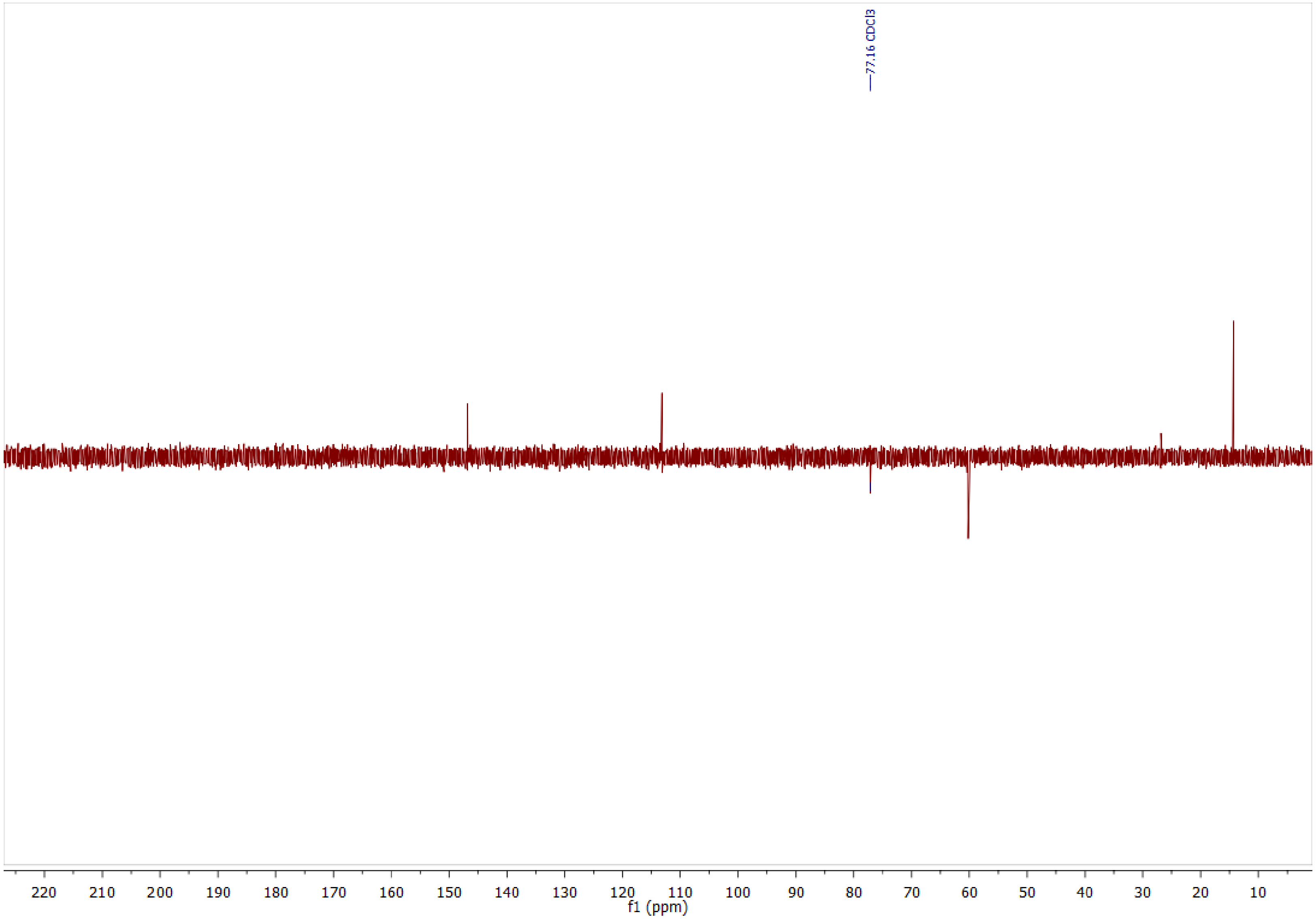

Figure S10: ^{13}C-DEPT spectrum ($CDCl_3$, 175 MHz) of (*E*)-**42**.
